# Open-source controller for low-cost and high-speed atomic force microscopy imaging of skin corneocyte nanotextures

**DOI:** 10.1016/j.ohx.2022.e00341

**Published:** 2022-07-25

**Authors:** Hsien-Shun Liao, Imtisal Akhtar, Christian Werner, Roman Slipets, Jorge Pereda, Jen-Hung Wang, Ellen Raun, Laura Olga Nørgaard, Frederikke Elisabet Dons, Edwin En Te Hwu

**Affiliations:** a Department of Mechanical Engineering, National Taiwan University, Taipei, Taiwan; b The Danish National Research Foundation and Villum Foundation's Center for Intelligent Drug Delivery and Sensing Using Microcontainers and Nanomechanics, Department of Health Technology, Technical University of Denmark, Lyngby, Denmark; c Physikalisch-Technische Bundesanstalt, Bundesallee 100, 38116 Braunschweig, Germany; d Department of Mechatronics and Robotics, Technical University of Munich, Germany

**Keywords:** High-speed atomic force microscopy, Sinusoidal scanning, Nanotexture, Corneocyte, Skin barrier function, AFM, atomic force microscope, AVT, anti-vibration table, FES, focus error signal, FPGA, field-programmable gate array, HS-AFM, high-speed atomic force microscope, OPU, optical pick-up unit, PC, personal computer, RIBM, Research Institute of Biomolecule Metrology, VCM, voice coil motor

## Abstract

High-speed atomic force microscopes (HS-AFMs) with high temporal resolution enable dynamic phenomena to be visualized at nanoscale resolution. However, HS-AFMs are more complex and costlier than conventional AFMs, and particulars of an open-source HS-AFM controller have not been published before. These high entry barriers hinder the popularization of HS-AFMs in both academic and industrial applications. In addition, HS-AFMs generally have a small imaging area that limits the fields of implementation. This study presents an open-source controller that enables a low-cost simplified AFM to achieve a maximum tip-sample velocity of 5,093 µm/s (9.3 s/frame, 512 × 512 pixels), which is nearly 100 times higher than that of the original controller. Moreover, the proposed controller doubles the imaging area to 46.3 × 46.3 µm^2^ compared to that of the original system. The low-cost HS-AFM can successfully assess the severity of atopic dermatitis (AD) by measuring the nanotexture of human skin corneocytes in constant height DC mode. The open-source controller-based HS-AFM system costs less than $4,000, which provides resource-limited research institutes with affordable access to high-throughput nanoscale imaging to further expand the HS-AFM research community.

## Highlights

• An open-source controller is proposed for a high-speed an atomic force microscope.
• The AFM has a maximum tip-sample velocity of 2,546.5 µm/s (9.3 s/frame)
• The scanning velocity is 100 times higher than that of the original controller.
• The HS-AFM has a resolution of 512 × 512 pixels and imaging area of 46.3 × 46.3 µm.
• The low-cost HS-AFM can measure the nanotexture of human skin corneocytes.

## Graphical abstract

**Figure:**
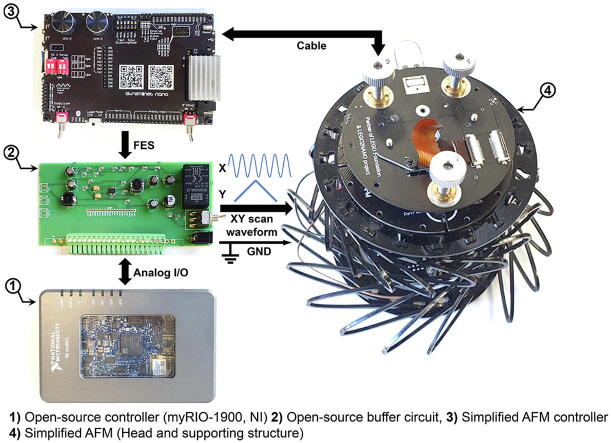

**Specification table:** 

Hardware name	OPEN-SOURCE HIGH-SPEED ATOMIC FORCE MICROSCOPE CONTROLLER
Subject area	• Engineering and materials science • Low-cost alternative • Hardware modifications to existing infrastructure • Biological sciences
Hardware type	• Imaging tool • Measuring physical properties and in-lab sensors • Electrical engineering • Mechanical engineering and materials science
Closest commercial analog	Conventional atomic force microscopes
Open-source license	*CC BY-SA 4.0*
Cost of hardware	*3,963 USD*
Source file repository	https://osf.io/wgx4p/

## 1 Hardware in context

High-resolution imaging systems, such as atomic force microscopes (AFMs) [1], [2], scanning electron microscope [3], [4], nonlinear optical microscope [5], [6], Raman microscope [7], [8], and confocal microscope [9], [10], require a raster scanning mechanism to achieve nanoscale and even atomic-scale resolution imaging. AFM is prominent among these systems, owing to its advantages of atomic resolution, quantitative 3D imaging, and usability in vacuum [11], [12], ambient [13], [14], [15], and liquid environments [16], [17], [18], [19]. Therefore, AFMs have been widely utilized in various disciplines, such as physics [20], semiconductor technology [21], nano-metrology [22], biological studies [23], [24], polymer chemistry [25], [26], molecular interaction [27], and dermatology [28]. However, raster scanning is time-consuming, and conventional AFMs typically require several minutes to capture a single image.

Efforts to increase the imaging speed of AFMs were undertaken in the 1990s [29], [30]. By improving the working bandwidth of the mechanical and electrical components, research groups developed custom high-speed atomic force microscopes (HS-AFMs) with high imaging temporal resolution to explore dynamic phenomena at the nanoscale [31], [32]. For example, the current HS-AFMs are capable of video-rate imaging for observing dynamic biomolecular phenomena [33], [34], [35], [36], [37]. However, HS-AFMs are still not commercially popular because they are highly complex and require dedicated personnel to operate. Moreover, the high-bandwidth mechanical and electrical hardware make the HS-AFMs even more expensive than conventional AFMs. For instance, the Research Institute of Biomolecule Metrology (RIBM) produced a video-rate AFM solution with a price of up to hundrds of thousand US dollars [38], [39]. Another disadvantage of existing HS-AFM systems is that they have closed-source software/hardware, and researchers cannot easily access or modify internal components and functions for their studies.

Several projects have focused on building low-cost AFMs that can be used for hands-on nanotechnology educational purposes. Loh et al. demonstrated an AFM costing $12,300 for undergraduate laboratories [40]. This AFM can achieve a scan rate of 0.14 lines/s (18 min/frame for 150 × 150 pixels), with lateral and vertical resolutions of up to 20 nm. Bergmann constructed a low-cost AFM setup for undergraduate and secondary school students [41] that costs more than $10,000. Grey described a low-cost simplified AFM built by a LEGO2NANO project that exceeds the requirements of standard lessons [42], [43], [44]. These low-cost AFM systems generally have low scanning speeds. However, we discovered that one commercial low-cost simplified AFM (Strømlingo DIY AFM, Strømlinet Nano) has a scanner with a resonance frequency of 55 Hz, and therefore has the potential for high-speed scanning. Thus, we developed an open-source controller for this simplified AFM.

This paper presents an open-source controller that enables a commercial low-cost simplified AFM system to be used for high-speed imaging. To the best of our knowledge, open-source HS-AFM controller hardware and/or software have not been published before. The proposed open-source controller improves the scan speed of the simplified AFM from 0.6 to 55 lines/s. Experimental results show that the proposed HS-AFM controller also improves both the scanning area and pixel size compared to that of the original controller of the simplified AFM. Furthermore, the proposed low-cost HS-AFM based on the open-source controller can successfully assess skin barrier function by measuring the nanotexture of human skin corneocytes in constant height DC mode. This study aims to expand the HS-AFM research community by lowering the price threshold of HS-AFM research and demonstrating a biomedical application that can be achieved without complex feedback control algorithms.

## 2 Hardware description

**Fig. 1:**
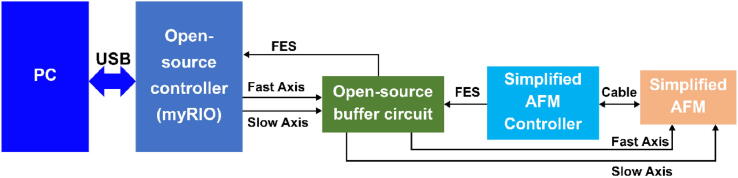
Block diagram of the low-cost HS-AFM system based on the open-source controller. A focus error signal (FES) is calculated by the simplified atomic force microscope (AFM) controller.

Fig. 1 presents a block diagram of the HS-AFM based on the open-source controller. This controller contains an open-source buffer circuit and an embedded device (myRIO-1900, National Instruments) with open-source control software. The controller connects to a commercial simplified AFM system (Strømlingo DIY AFM, Strømlinet Nano), which includes an Arduino-based controller and AFM head on a supporting structure. The open-source controller generates fast-axis (FastX) and slow-axis (SlowY) scanning signals through two analog outputs, and one analog input receives a focus error signal (FES) [45] generated by the simplified AFM controller. The scan data are transferred from the HS-AFM controller to a personal computer (PC) via the first-in-first-out (FIFO) queuing mechanism to display the real-time scanning image.

### 2.1 Open-source controller

The embedded device of the controller is based on a field-programmable gate array (FPGA) real-time microcontroller unit, which enables high-speed scanning control and parallel function execution. Moreover, the FPGA can be programmed using the LabVIEW software (National Instruments) with a user-friendly graphical interface. In addition, the open-source LabVIEW code of the controller provides a foundation on which users are able to build their own functions. The embedded device has a mini system port (MSP) terminal that interfaces the digital-to-analog converters (DACs) and analog-to-digital converters (ADCs). Two of these DACs (AO0 and AO1), with 12 bits resolution and 345 kS/s update rates, are used to output FastX and SlowY driving signals, respectively. The FES is connected to one ADC, with a resolution of 12 bits and a sampling rate of 500 kS/s.

**Fig. 2:**
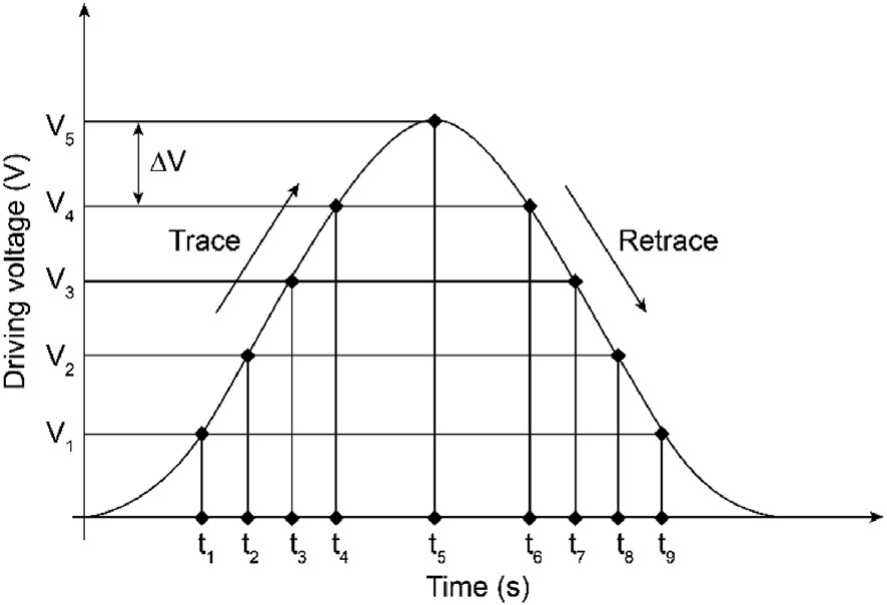
Data sampling method for the sinusoidal scanning motion.

To avoid unwanted scanner oscillation excited by conventional zig-zag raster scanning, a different approach was followed to implement the open-source HS-AFM. The fast-axis is driven by a sinusoidal scanning signal to eliminate high-frequency components when the scanning direction changes. The sinusoidal signal is generated by the open-source controller using a lookup table for high-speed scanning. A drawback of using the sinusoidal trajectory is that the velocity of the sinusoidal scanning motion is not constant, which can cause image distortion while using a constant sampling rate. To solve this problem, the HS-AFM controller captures the FES using a non-constant sampling rate, as illustrated in Fig. 2. The FES is acquired when the sinusoidal scanning signal arrives at defined voltage levels with a constant voltage interval Δ*V*. The scanning signal is proportional to the scanner displacement if we assume the time delay between the driving signal and the movement to be negligible. Therefore, an FES with a constant displacement interval can be obtained using a time-varying sampling rate.

**Fig. 3:**
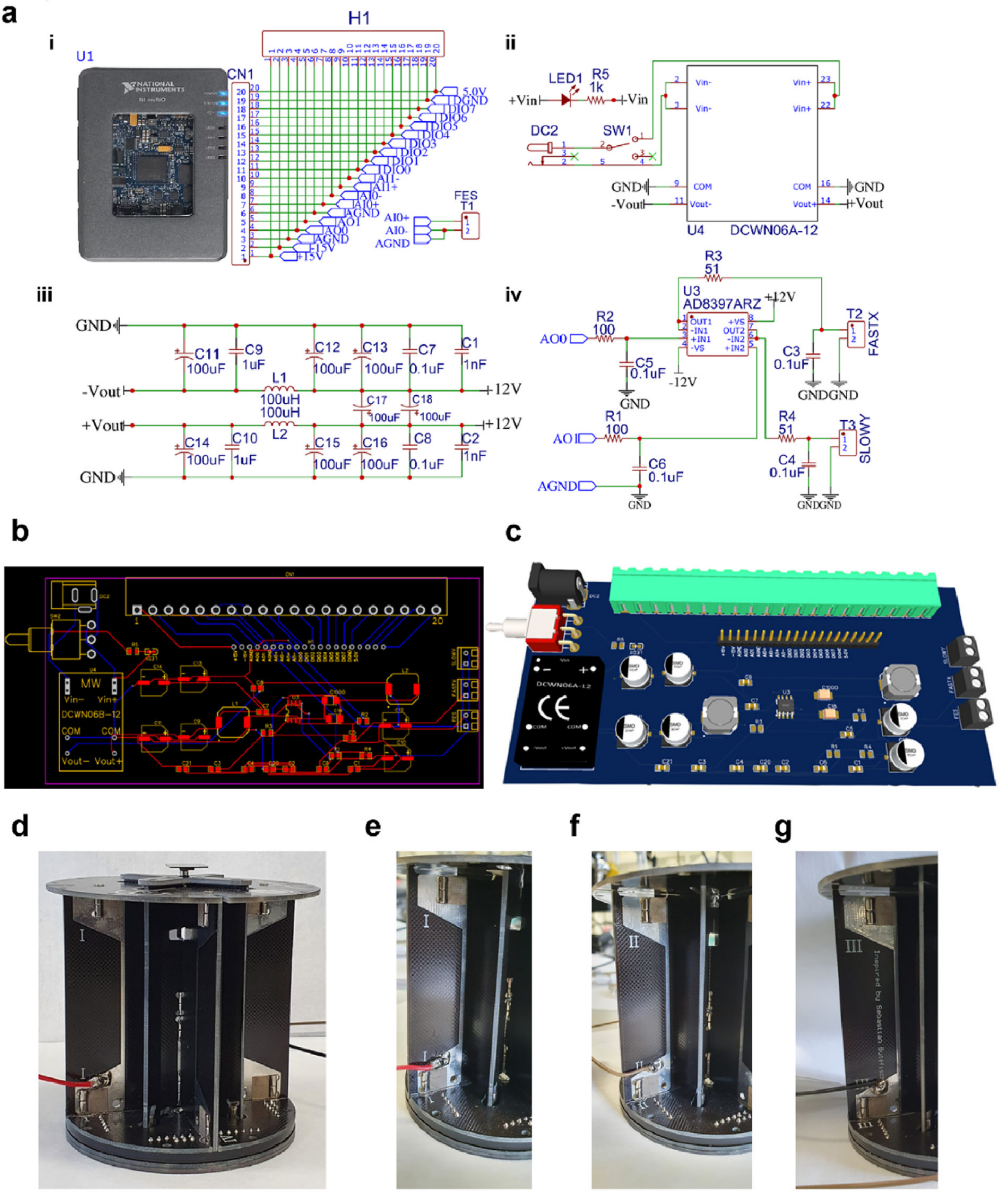
Open-source controller connections and schematic with the open-source buffer circuit. **a) i)** Open-source controller connections with external I/O, **ii)** 12 V DC-DC converter, **iii)** network of capacitors and inductors to reduce the noise at the DC-DC converter output, and **iv)** schematic of the buffer circuit. **b)** PCB schematic and **c)** 3D view of the open-source buffer circuit. **d)** Image of the AFM base. A sample stage is located on top of the base. **e–g)** Wire connections from point I, II, and III soldered to the open-source buffer circuit for FastX, SlowY, and GND, respectively.

**Fig. 4:**
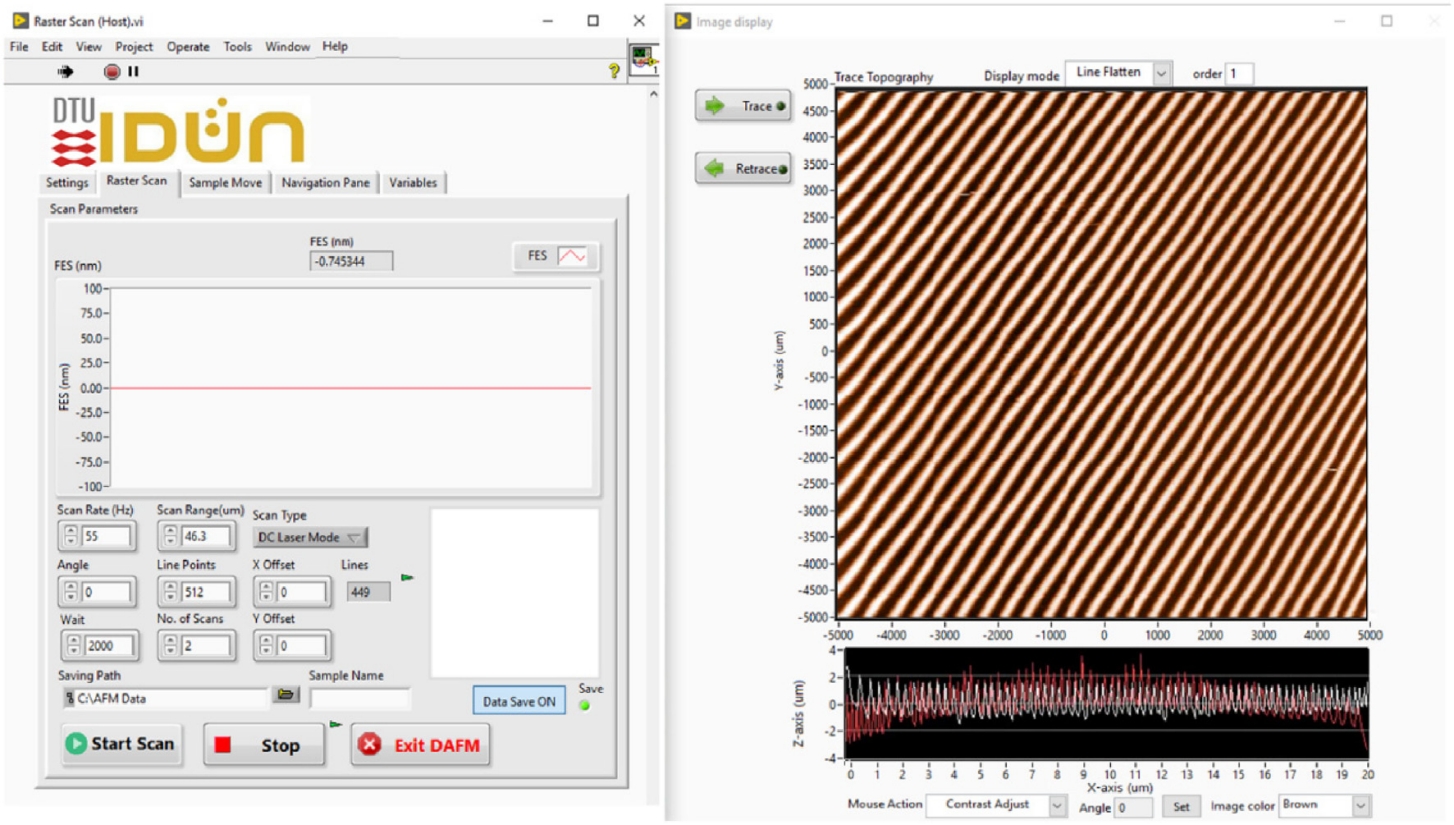
Control and imaging windows of the user interface of the HS-AFM controller.

**Fig. 5:**
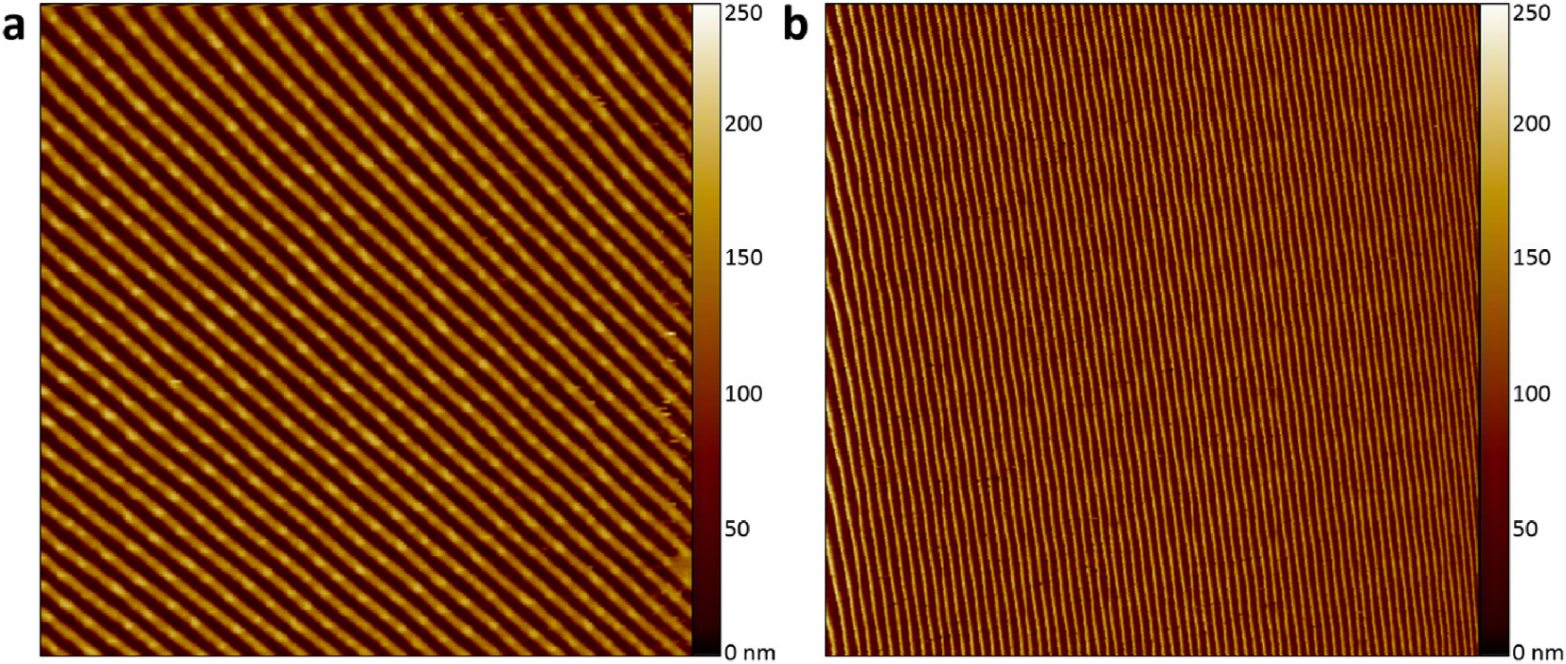
Comparison of the full scanning area of the different controllers. AFM image of DVD tracks using **a)** the simplified AFM controller (area: 23 × 23 µm^2^) and **b)** the open-source HS-AFM controller (area: 46.3 × 46.3 µm^2^). The slight image distortion on the left is due to the hysteresis of the scanner.

### 2.2 Open-Source buffer circuit

The open-source buffer circuit is built to amplify the FastX and SlowY scanning signals to overcome the output current limitation (2 mA) of the embedded device. The buffer circuit consists of an operational amplifier (AD8397ARZ, Analog Devices), which supplies the FastX and SlowY driving signals with a maximum output current and voltage of 310 mA and ±12 V, respectively. The amplified driving signals are directly connected to the scanner of the simplified AFM for high-speed scanning, as shown in Fig. 1. The buffer circuit has a connector that directly plugs into a mini system port of the embedded device.

### 2.3 Simplified AFM

The simplified AFM consists of a controller, an AFM head, a supporting structure that contains an AFM base, and an anti-vibration device. The simplified AFM controller is connected to the AFM head via a flat cable. The AFM head utilizes a DVD optical pick-up unit (OPU) [46], [47], [48] to monitor the cantilever deflection [49], [50], [51] of the AFM probe [52], [53], [54]. The AFM base consists of a piezoelectric buzzer-based scanner [55], [56] to scan a sample in the x- and y-directions. The anti-vibration device is a cage-like flexible structure that supports the AFM base and isolates the external vibration during measurement.

The simplified AFM controller has two knobs, VCM_Z and VCM_X, to control the movement in the z- and x-directions of the voice coil motor (VCM) on the OPU. The VCM actuates an objective lens of the OPU to focus a laser on the AFM probe. The simplified AFM controller calculates signals from the OPU [57] and provides the FES that is proportional to the AFM probe deflection [58]. The FES detection range can be adjusted to 1 µm, 2 µm, or 4 µm using dip switches on the controller, and the corresponding resolutions are 0.2 nm, 0.48 nm, and 0.97 nm, respectively. The simplified AFM has a maximum scan speed of 0.6 lines/s (see the video “Simplified AFM with the original controller for 0.6 lines per second imaging.mp4″) with a limited image resolution of 256 × 256 pixels and a small scan range of 23 × 23 µm^2^. The controller provides signal input pins for external scanning control. However, buffer circuits inside the controller cannot provide sufficient current output, and the maximum scanning speed is limited to 13.8 µm/s. Thus, the proposed approach is to bypass the original controller and connect the scanning signals in the x- and y-directions to the AFM scanner directly.

In summary, the presented open-source controller provides the following benefits:

• Enables a low-cost simplified system for HS-AFM imaging.
• Eliminates unwanted scanner oscillation with sinusoidal scanning motion.
• Achieves 5,093 µm/s tip-sample velocity with a constant-height DC mode.
• Offers a wide imaging area of 46.3 × 46.3 µm^2^ with 512 × 512 pixel size.
• Uses open-source LabVIEW code as a foundation for further modifications.
• Allows high-speed corneocyte nanotexture imaging.

## 3 Design file summary

**Table:** 

**Design file name**	**File type**	**Open-source license**	**File location**
Fig. 1	Figure	CC BY-SA 4.0	Available at https://osf.io/wgx4p/
Fig. 2	Figure	CC BY-SA 4.0	Available at https://osf.io/wgx4p/
Fig. 3	Figure	CC BY-SA 4.0	Available at https://osf.io/wgx4p/
Fig. 4	Figure	CC BY-SA 4.0	Available at https://osf.io/wgx4p/
Fig. 5	Figure	CC BY-SA 4.0	Available at https://osf.io/wgx4p/
Graphical abstract	Figure	CC BY-SA 4.0	Available at https://osf.io/wgx4p/
LabVIEW source code	Code	CC BY-SA 4.0	Available at https://osf.io/wgx4p/
LabVIEW exe file	Executable file	CC BY-SA 4.0	Available at https://osf.io/wgx4p/
Buffer circuit Ver. 1.0 design	Design file	CC BY-SA 4.0	Available at https://osf.io/wgx4p/
Buffer circuit Ver. 2.0 design	Design file	CC BY-SA 4.0	Available at https://osf.io/wgx4p/
Simplified AFM with the original controller for 0.6 lines per second imaging	Video (Mp4)	CC BY-SA 4.0	Available at https://osf.io/wgx4p/
Simplified AFM with the open-source controller for 55 lines per second imaging	Video (Mp4)	CC BY-SA 4.0	Available at https://osf.io/wgx4p/
Operation process of the open-source controller-based HS-AFM	Video (Mp4)	CC BY-SA 4.0	Available at https://osf.io/wgx4p/
Simplified AFM scanner calibration process	Video (Mp4)	CC BY-SA 4.0	Available at https://osf.io/wgx4p/
Installation of myRIO for the open-source controller	Video (Mp4)	CC BY-SA 4.0	Available at https://osf.io/wgx4p/
Focus laser on an AFM probe and start measurement	Video (Mp4)	CC BY-SA 4.0	Available at https://osf.io/wgx4p/

### 4 Bill of materials summary

**Table:** 

**Designator**	**Component**	**Number**	**Cost per unit currency (USD)**	**Total cost (USD)**	**Source of materials**	**Material type**
U1	NI myRIO-1900	1	700	700	National Instruments Link: https://www.ni.com/da-dk/shop/hardware/products/myrio-student-embedded-device.html	Electronics
U2	Strømlinet DIY AFM AFM optomechanical system and controller Anti-vibration table	1 1	2,999 199	3,198	Strømlinet Link: https://www.stromlinet-nano.org/str%C3%B8mlingo-nssembly-afm	Electromechanical system
U3	AD8397ARZ	1	3.56	3.56	Digikey Link: https://dk.rs-online.com/web/p/operationsforstaerkere/7097118	Electronics
U4	DC-DC converter 12 V	1	10.7	10.7	Digikey Link: https://www.digikey.dk/products/da?keywords = DCWN06A-12	Electronics
R1, R2	Resistor 100 Ω	2	0.04	0.08	Digikey Link: https://www.digikey.dk/product-detail/da/susumu/RG2012P-101-B-T5/RG20P100BTR-ND/1240651	Electronics
*R*3, R4	Resistor 51 Ω	2	0.1	0.2	Digikey Link: https://www.digikey.dk/product-detail/da/panasonic-electronic-components/ERA-6AED510V/P123854CT-ND/9467783	Electronics
R5	Resistor 2.2 kΩ	1	0.04	0.04	Digikey Link: https://www.digikey.dk/product-detail/da/panasonic-electronic-components/ERJ-1TYJ222U/PT2-2KXTR-ND/365168	Electronics
LED1	LED	1	0.04	0.04	Digikey Link: https://www.digikey.dk/product-detail/da/lite-on-Inc/LTST-C191KFKT/160–1445-2-ND/386833	Electronics
C1, *C*2	Capacitor 1 nF	2	0.11	0.22	Digikey Link: https://www.digikey.dk/product-detail/da/avx-corporation/08055C102KAT2A/478–1371-1-ND/564403	Electronics
C3-C8	Capacitor 0.1 μF	4	0.1	0.4	Digikey Link: https://www.digikey.dk/product-detail/da/avx-corporation/08055C104KAT4A/478–10836-1-ND/7536355	Electronics
C9, C10	Capacitor 1 μF	2	0.1	0.2	Digikey Link: https://www.digikey.dk/product-detail/da/samsung-electro-mechanics/CL21B105KOFNNNE/1276–1026-1-ND/3889112	Electronics
C11-C18	Capacitor 100 μF	6	3.09	18.54	RS Link: https://dk.rs-online.com/web/p/aluminium-kondensatorer/7111441	Electronics
L1, L2	Inductor 100 μH	2	1.34	2.68	Digikey Link: https://www.digikey.dk/product-detail/da/w%C3%BCrth-elektronik/7447714101/732–2989-2-ND/2625928	Electronics
DC1	DC power adapter 12 V	1	8.85	8.85	Digikey Link: https://www.digikey.dk/product-detail/da/xp-power/VER05US120-JA/1470–2781-ND/5023723	Electronics
DC2	DC power jack	1	0.58	0.58	Digikey Link: https://www.digikey.dk/product-detail/da/cui-devices/PJ-037A/CP-037A-ND/1644545	Electronics
SW1	SPDT toggle switch	1	5.23	5.23	Digikey Link: https://www.digikey.dk/products/da?keywords = M2013S2A2W30	Electronics
T1-T3	Terminal blocks	3	0.53	1.59	Digikey Link: https://www.digikey.dk/product-detail/da/phoenix-contact/1935776/277–6405-ND/2513905	Electronics
CN1	Pluggable terminal blocks	1	10.10	10.10	Mouser Link: https://www.mouser.dk/ProductDetail/Phoenix-Contact/1840353?qs=aYsvlkyO7qONsQFTToFdhQ%3D%3D	Electronics
H1	Male header	1	1.64	1.64	Digikey Link: https://www.digikey.dk/product-detail/da/sullins-connector-solutions/PPTC101LFBN-RC/S7008-ND/810149	Electronics

## 5 Building instructions

### 5.1 Tools

The tools required are as follows:

• Soldering iron and solder
• Wire stripper
• Screwdriver (3 mm)

### 5.2 Building procedure

1. Connect the AFM head to the simplified AFM controller with a flexible flat cable.
2. Connect the open-source controller to a PC using a USB cable (type B to type A).
3. Connect the open-source buffer circuit to the open-source controller.
4. Solder three wires at the AFM base at positions marked by Roman numerals I (FastX), II (SlowY), and III (GND), as shown in Fig. 3.
5. Connect wires I and II from the simplified AFM to the terminal blocks of the open-source buffer circuit labeled FastX and SlowY, respectively. Wire III from the simplified AFM can be connected to either FastX or SlowY GND because the ground is commonly connected.
6. Connect the FES and GND of the simplified AFM controller to the terminal block of the open-source buffer circuit labeled FES and GND.
7. Connect DC power adapters to the open-source controller, open-source buffer circuit, and simplified AFM controller. Do not turn on the power before all cables are appropriately connected.

## 6 Operation instructions

The user interface was developed in LabVIEW and executed on a PC. The following procedure should be followed to operate the open-source controller-based HS-AFM system.

1. Install the executable file (AFM.exe) on the PC.
2. Open the software installed in the PC directory (C drive is the default installation directory). Doing so opens the control and imaging windows, as shown in Fig. 4. The FES appears in the control window.
3. Install the AFM probe on the AFM head. The cantilever of the AFM probe should be positioned in front of the objective lens of the OPU [59].
4. Adjust the VCM_Z and VCM_X knobs on the simplified AFM controller to adjust the laser focus on the cantilever. Turning the VCM_Z knob back and forth produces an S-shaped curve in the FES of the control window. Adjust the position of VCM_Z such that the FES is in the linear range of the S-curve.
5. A video in the file repository entitled “Focus laser on an AFM probe and start measurement.mp4″ demonstrates the laser alignment process in detail.
6. Load a sample on the AFM sample stage and mount the AFM head on the AFM base such that the three precision screws are in contact with the AFM base. Ensure sufficient space between the AFM probe and sample before mounting the AFM head on the AFM base.
7. To test the sensitivity of the system, gently tap on one of the screws; the variation in the FES is displayed in the control window.
8. Carefully approach the AFM probe by turning the three precision screws equally to avoid tilting the AFM head. Because the approaching mechanism is a manual process, setpoint and feedback control are unnecessary. While approaching the AFM probe, the oscillation of the FES can be seen in the control window; the amplitude becomes constant once the tip is in contact with the sample. When the change in the FES amplitude is more than 20 %, stop the approaching process.
9. Define the scanning parameters (scan rate, range, type) and press the start scan button in the control window to start scanning. The image appears in the imaging window.
10. The image contrast can be adjusted by using the mouse and drawing a rectangle on the trace or retrace image.
11. A video in the file repository entitled “Operation process of the open-source controller-based HS-AFM.mp4″ demonstrates the software operation in detail.

## 7 Characterization and implementation

### 7.1 Calibration and limitation

A piece (ca. 1 × 1 cm^2^) of a data track layer (from a rewritable DVD) was used as a low-cost sample to calibrate the HS-AFM scanner. The DVD data tracks have a fixed period of 740 nm and a defined depth of 160 nm [60]. The scanning area and Z sensitivity of the FES can be calibrated by measuring the DVD data tracks. DC mode AFM probes with a spring constant of 0.03 N/m (aluminum coating on the detector side, Mikromasch, Germany) were used for nanoscale imaging. The HS-AFM calibration was performed in constant-height DC mode in an ambient environment. The HS-AFM image was subjected to raw data processing with free data analysis software (Gwyddion [61]). A video in the file repository entitled “Simplified AFM scanner calibration process.mp4″ shows the calibration process in detail.

The performances of the simplified AFM and open-source HS-AFM controllers were compared experimentally. Fig. 5a and 5b show the images of the DVD tracks acquired by the two controllers with the same AFM head and supporting structure. The simplified AFM controller has a maximum scanning speed of 0.6 lines/s and an image display resolution of 256 × 256 pixels. It takes 426.6 s (7 min) to complete one measurement. The maximum measurement area is 23 × 23 µm^2^; one pixel equals 89.8 × 89.8 nm^2^. In comparison, the open-source HS-AFM controller provides a scanning speed 91.6-fold faster (55 lines/s) and a higher image resolution of 512 × 512 pixels. One image takes 9.3 s to acquire, which is 45.8 times faster than the simplified AFM controller. However, the HS-AFM controller has a slightly lower resolution per pixel, 90.4 × 90.4 nm^2^, owing to the larger measurement area (46.3 × 46.3 µm^2^). The respective imaging processes of the two controllers are shown in two videos in the file repository entitled “Simplified AFM with the original controller for 0.6 lines per second imaging.mp4″ and ”Simplified AFM with the open-source controller for 55 lines per second imaging.mp4″.

Three main parameters are responsible for limiting the maximum imaging speed of the HS-AFM. First, the DACs of the embedded device can provide a maximum scanning rate of 336 lines/s (345 kS/s update rate divided by trace/retrace direction in a total of 1024 points). Second, the current output from the open-source buffer circuit is 310 mA, which can drive the scanner capacitive load (single axis: 72 nF) for scanning at more than 1,000 lines/s. Lastly, the buzzer scanner has a resonance frequency of 55 Hz, which is a bottleneck in terms of increasing the scanning speed in this study. In other words, we expect the imaging speed of the HS-AFM based on the open-source controller to increase by increasing the resonance frequency of the scanner.

### 7.2 Nanotexture based skin barrier function assessment

Clinical studies confirmed that the density of circular nanotextures (typical height: 273 nm, width: 305 nm) on human corneocyte surfaces is inversely associated with natural moisturizing factor (NMF) concentrations [62]. Moreover, an image recognition algorithm based on machine learning was developed to quantify the circular nanotexture density within 20x20 µm^2^ areas into a dermal texture index (DTI) [63]. The DTI could be an objective score to assess the severity of atopic dermatitis (AD) [64]. The DTI score ranges from 0 to 800. Healthy skin has a score of less than 100, and a score of 200 is the threshold for AD clinical symptoms. A score over 400 indicates that a severe case of AD is expected.

**Fig. 6:**
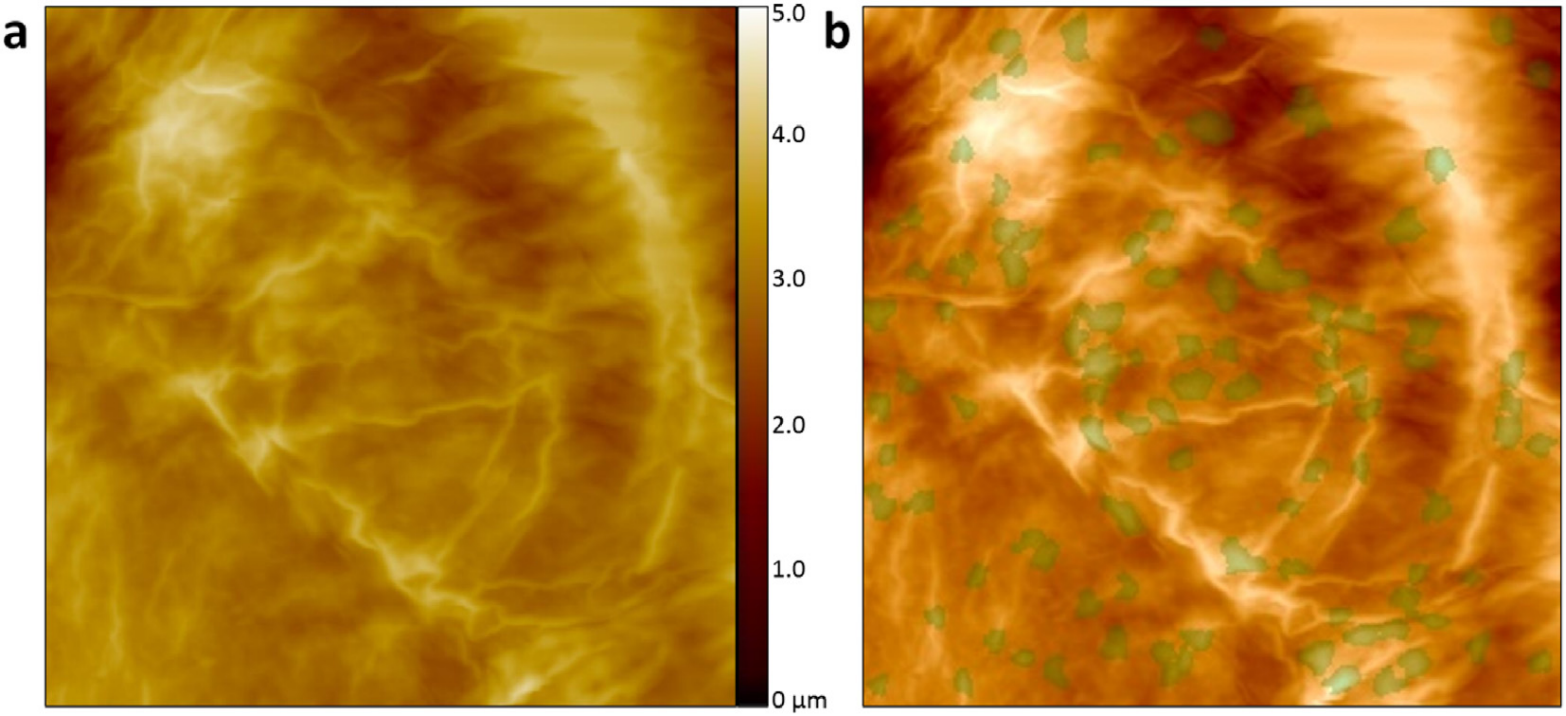
Quantification of skin corneocyte surface nanotextures. **a)** Surface topography of skin corneocytes (area: 20 × 20 µm^2^) acquired by the open-source controller-based HS-AFM in constant height DC mode. **b)** The 135 circular nanotextures recognized and labeled by the evaluation method based on machine learning. Thus, the DTI score is 135.

The HS-AFM based on the open-source controller is capable of assessing skin barrier function by imaging nanotextures on human skin corneocyte surfaces. Because AFM probes that operate in DC mode have a low spring constant, the constant height DC scanning mode can effectively image the nanotextures on skin corneocytes. Fig. 6a shows a 20 × 20 µm^2^ topography image of a skin corneocyte from a healthy donor. An evaluation method based on machine learning, DERMATACT (Serend-Ip GmbH, Münster, Germany), was used to analyze this image, and 135 circular nanotextures (indicated in green) were recognized, as shown in Fig. 6b. The total area and the average height of the circular nanotextures are 4.22 × 10^7^ ± 2.9 × 10^6^ nm^2^ and 297 ± 20 nm, respectively.

**Fig. 7:**
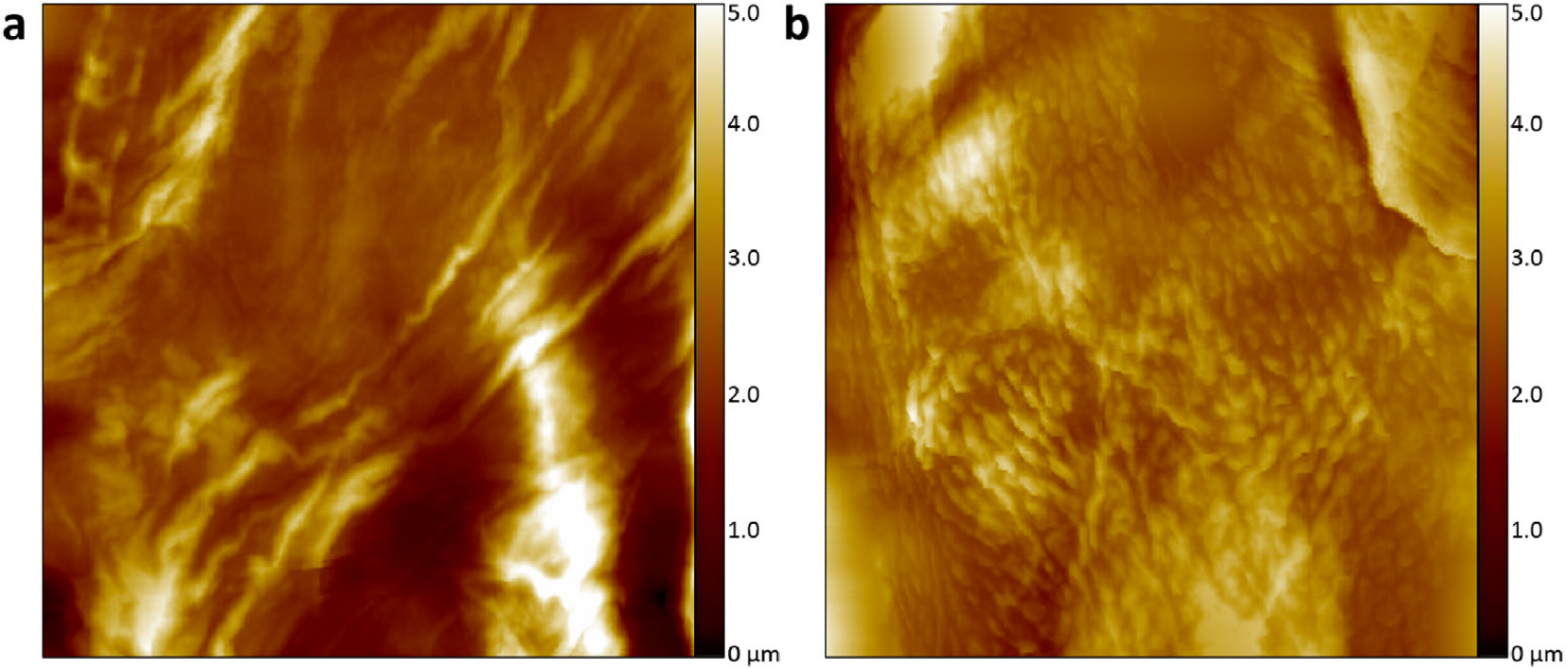
Surface nanotexture of healthy and AD lesional skin corneocytes acquired by the open-source controller-based HS-AFM. Surface topography of **a)** a healthy control corneocyte (area: 20 × 20 µm^2^) with a DTI of 65. **b)** an AD lesional skin corneocyte (area: 20 × 20 µm^2^) with a DTI of 332.

The HS-AFM based on the open-source controller was subsequently used to examine healthy control and AD lesional skin corneocytes from donors. The healthy control yields a low DTI score of 65, as shown in Fig. 7a. The AD lesional skin (Fig. 7b) has many circular nanotextures and the DTI score is 332. The results show that the proposed open-source HS-AFM can successfully differentiate healthy and AD skins.

## 8 Conclusion

The open-source controller transforms the existing infrastructure, a low-cost simplified AFM, into a high-speed AFM (HS-AFM) with a tip-sample velocity of 5,093 µm/s. Moreover, the HS-AFM acquired one skin nanotexture image in 9.3 s with a constant height DC mode. The acquired skin nanotexture images satisfy the DTI calculation requirements, scan area: 20 × 20 µm^2^ and image pixel: 512 × 512 pixels, for quantified AD severity assessment. The nanotexture images, when combined with DTI scores, can be used to differentiate between healthy and AD skins. The open-source controller fulfills the need for samples with a large area and flat morphology (such as skin corneocytes with a height difference <3 µm) with constant-height DC mode HS-AFM measurement. We believe that in addition to working on simplified AFMs, the open-source controller can upgrade old AFMs to have high-speed DC mode measurements, thereby further expanding the HS-AFM research community.

## 9 Potential modifications

To optimize the performance of the open-source buffer circuit, we added 1 Ω resistors to dampen the resonance frequency of the power supply filter. Buffer capacitors were added to stabilize the operational amplifier. The modified open-source buffer circuit design files can be found in the file repository entitled “buffer circuit Ver. 2.0 design”. In the buffer circuit Ver. 2.0 design, FastX and SlowY are assigned the labels FASTXOUT and SLOWYOUT, respectively.

## Ethics statement

Informed and written consents were obtained from all participants, and the study was conducted in accordance with the Declaration of Helsinki principles. The protocol was approved by the regional ethics committee (H-2207232) and the Danish Data Protection Agency.

## CRediT authorship contribution statement

**Hsien-Shun Liao:** Writing – original draft, Methodology, Validation, Software. **Imtisal Akhtar:** Writing – original draft, Investigation, Visualization. **Christian Werner:** Investigation, Methodology, Formal analysis, Visualization. **Roman Slipets:** Investigation, Formal analysis. **Jorge Pereda:** Writing – review & editing. **Jen-Hung Wang:** Formal analysis, Visualization. **Ellen Raun:** Investigation, Validation. **Laura Olga Nørgaard:** Investigation, Data curation. **Frederikke Elisabet Dons:** Investigation. **Edwin En Te Hwu:** Conceptualization, Methodology, Supervision, Data curation, Writing – review & editing.

## Declaration of Competing Interest

The authors declare the following financial interests/personal relationships which may be considered as potential competing interests: The author Dr. En-Te Hwu was a technical consultant of the simplified AFM company (Strømlinet Nano). This does not affect his adherence to scientific standards.
